# Control and Effort Costs Influence the Motivational Consequences of Choice

**DOI:** 10.3389/fpsyg.2017.00675

**Published:** 2017-05-03

**Authors:** Holly Sullivan-Toole, John A. Richey, Elizabeth Tricomi

**Affiliations:** ^1^Graduate Program in Translational Biology, Medicine and Health, Virginia Tech, BlacksburgVA, USA; ^2^Psychology Department, Virginia Tech, BlacksburgVA, USA; ^3^Psychology Department, Rutgers University-Newark, NewarkNJ, USA

**Keywords:** choice, personal control, effort, motivation, decision-making, perceived control

## Abstract

The act of making a choice, apart from any outcomes the choice may yield, has, paradoxically, been linked to both the enhancement and the detriment of intrinsic motivation. Research has implicated two factors in potentially mediating these contradictory effects: the personal control conferred by a choice and the costs associated with a choice. Across four experiments, utilizing a physical effort task disguised as a simple video game, we systematically varied costs across two levels of physical effort requirements (Low-Requirement, High-Requirement) and control over effort costs across three levels of choice (Free-Choice, Restricted-Choice, and No-Choice) to disambiguate how these factors affect the motivational consequences of choosing within an effortful task. Together, our results indicated that, in the face of effort requirements, illusory control alone may not sufficiently enhance perceptions of personal control to boost intrinsic motivation; rather, the experience of actual control may be necessary to overcome effort costs and elevate performance. Additionally, we demonstrated that conditions of illusory control, while otherwise unmotivating, can through association with the experience of free-choice, be transformed to have a positive effect on motivation.

## Introduction

People will fight for their right to choose in some instances and flip a coin to avoid choosing in others; in parallel, research on the relationship between choice and motivation is complex and has produced results that are often conflicting. Research on the motivational consequences of choosing, rather than focusing on the content of choices, has focused on how the very act of making a choice influences valuation processes and intrinsic motivation—and the findings have been contradictory. The act of making a choice, separable from any extrinsic gains or losses the decision may incur, has been linked to both motivational enhancements and decrements (Botti and Iyengar, 2004; Patall et al., 2008; Patall, 2012).

From these paradoxical findings, however, two major factors have emerged as potential mediators of whether the act of choosing has a positive or negative effect on intrinsic motivation: (1) the personal control provided by a choice and (2) the costs associated with making a choice (Patall, 2012). Thus, the utility of making a choice, in terms of its effect on motivation, may be recast as a joint function of the control provided by and costs associated with that choice. Yet, little is known about how control and cost, separately and conjointly, influence the motivational effects of the act of choosing. Accordingly, the purpose of the current study is to disambiguate how control and cost affect the motivational consequences of choosing within an effortful task.

There is substantial evidence that the act of making choices, in and of itself, is intrinsically rewarding and motivating (Leotti et al., 2010, 2015). For example, there is a measurable preference for options that lead to a subsequent, additional choice over options leading to a forced-choice, even when there are no material differences in outcomes (Suzuki, 1997, 2000; Bown et al., 2003). Neuroimaging studies have demonstrated that free-choices, although they bestow no additional extrinsic reward, enhance neural activation in value-related regions, both when anticipating a choice (Leotti and Delgado, 2011; Fujiwara et al., 2013) and when evaluating outcomes linked to choice (Tricomi et al., 2004; Cockburn et al., 2014). These studies suggest that intrinsic value is assigned to the very process of active decision making. Furthermore, engaging in active decision making can confer a variety of performance-related benefits, facilitating intrinsic motivation, performance, and effort exertion (Patall et al., 2008; Bhanji and Delgado, 2014; Murayama et al., 2015).

Conversely, there are also certain contexts in which passivity is valued and choice is avoided or appears to have a discounting effect on outcomes (Samuelson and Zeckhauser, 1988; Burger, 1989; Ritov and Baron, 1990; Anderson, 2003; Leotti and Delgado, 2014). This propensity for passive decision strategies suggests that avoiding decisions can also carry utility. This supposition, too, is supported by functional neuroimaging evidence demonstrating that passively maintaining a default option, rather than making an active decision, engaged the same neural region activated by winning money (Yu et al., 2010). Furthermore, in some circumstances, making choices may have deleterious effects on motivation and performance (Burger, 1989; Flowerday and Schraw, 2003; Botti and Iyengar, 2004; Vohs et al., 2008).

As choice can, depending on context, have a very different impact on intrinsic motivation and related outcomes, there is a need to identify contextual factors that mediate these effects. One potential mediating factor can be drawn from a frequent theme in psychological research: that perceptions of personal control are intrinsically motivating (Rotter, 1966; Rodin and Langer, 1977; Ryan and Deci, 2000; Leotti et al., 2010, 2015). Thus, it may not be the act of decision making *per se* that bestows psychological benefits but instead the sense of personal control conferred by making a decision. Consistent with this proposition, evidence suggests that the degree of personal control offered by a decision may mediate between its beneficial versus detrimental effects on intrinsic motivation (Katz and Assor, 2006; Patall, 2012). Further, studies that have dissociated perceptions of control from decision making scenarios have demonstrated that choices engendering perceptions of control, rather than the mere act of choice, were linked to motivational benefits (Reeve et al., 2003; Moller et al., 2006). Thus, substantial evidence indicates exercising personal control through making choices enhances intrinsic motivation. However, very few studies have directly, empirically assessed the role of personal control on the motivational effects of decision making.

The cost/benefit analysis, a core concept from economics, suggests that the costs associated with making a decision are another potential mediator of the beneficial versus detrimental consequences of choosing (Patall, 2012). Effort is frequently cited as a principal cost in decision-making, and there is a well-demonstrated effort discounting effect, whereby effort decreases the utility of related outcomes (Botvinick et al., 2009; Kool et al., 2010; Kurniawan et al., 2010, 2013). Similarly, in the context of choice, there is evidence that as decision-related costs increase, the utility and positive effects of choice are undermined. Making choices under conditions of high costs—effort costs, a loss frame, or negative emotions—can attenuate the appeal of engaging in active choice (Samuelson and Zeckhauser, 1988; Beattie et al., 1994; Iyengar and Lepper, 2000; Fleming et al., 2010; Kool et al., 2010; Leotti and Delgado, 2014). Furthermore, making choices in a context of increased costs can give rise to negative consequences such as reduced satisfaction with outcomes, increased negative emotions, and diminished performance (Garbarino and Edell, 1997; Iyengar and Lepper, 2000; Botti and Iyengar, 2004; Gourville and Soman, 2005; Vohs et al., 2008; Hafner et al., 2016).

Given the conflicting evidence for choice’s utility in enhancing intrinsic motivation and the theoretical basis for control and cost to mediate between the beneficial and detrimental effects of choosing, the current study sought to dissociate the motivational consequences of these two factors by varying the level of control conferred by choices across different levels of effort costs. Specifically, we utilized two levels of physical effort costs (a high and low effort requirement; hereafter High-Requirement and Low-Requirement), and three levels of control over effort costs: real control (Free-Choice), illusory control (Restricted-Choice), and no control (No-Choice). We assessed the motivational outcomes of choice via preference and performance linked to the different conditions, as these measures are commonly utilized in the choice literature as a proxy for motivation (Patall et al., 2008). As the focus of the present investigation was the impact of control and cost on intrinsic motivation, our experimental task utilized performance feedback rather than extrinsic reinforcers such as monetary reward.

We had three overarching hypotheses across four experiments comprising this study. Based on effort discounting theory, we hypothesized that (I) intrinsic motivation and thus motivational outcomes would be generally enhanced for lower compared to higher effort requirement trials (Low-Requirement > High-Requirement). Based on evidence that perceptions of control have positive effects on motivation, we further hypothesized that (II) intrinsic motivation and thus motivational outcomes would improve as a function of the amount of control available (Free-Choice > Restricted-Choice > No-Choice). Finally, based on a combination of evidence suggesting that a context of high costs and low personal control may produce a particularly damaging coalition, we anticipated that preference for and performance on an effortful task should be undermined most severely at the junction of high effort costs and low personal control (i.e., no available choices). Thus, we hypothesized that (III) intrinsic motivation and thus motivational outcomes would be most strongly diminished when no choice is offered, but effort expenditures are high. Across four experiments, our approach to testing these hypotheses was to first examine the individual influences of cost (effort requirements) and control (choice conditions) and then combine these factors to test their conjoint effects on motivational outcomes.

## General Method

**FIGURE 1:**
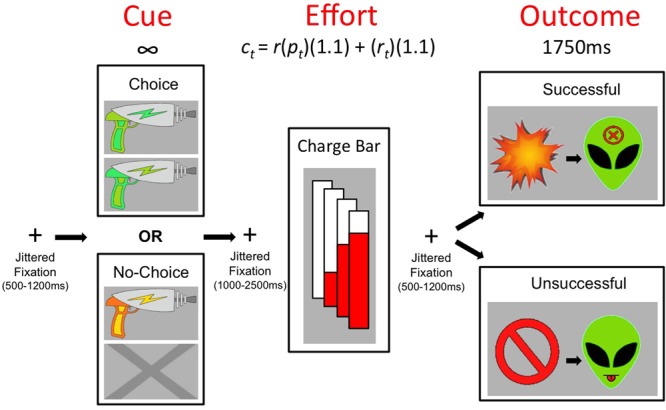
**Schematic of a single trial within the tasks.** During the cue period, a screen presented blaster options (two options for Choice or a single blaster for No-Choice) until the participant responded. When the “charge bar” appeared, participants began making fast, repetitive key presses to fill the bar according to the effort requirement. Time allotted on a given effort trial was determined by the formula *c_t_*= *r*(*p_t_*)(1.1) + (*r_t_*)(1.1), where c_t_ is allotted time, *r* is the required number of presses, and *r_t_* and *p_t_* are the participant’s average pre-game reaction time to make an initial key press and time between key presses, respectively. Outcomes indicating whether the charge bar was successfully filled in the given time were displayed for 1750 ms.

Participants were adult undergraduate students, recruited from Rutgers University-Newark, who provided written informed consent in accordance with the Declaration of Helsinki and were compensated with course credit. The Institutional Review Board of Rutgers University approved the study. To test study hypotheses, we created a novel physical effort task in E-Prime (Psychology Software Tools, Pittsburgh, PA, USA), which employed, in different combinations across four experiments, three choice conditions offering different levels of control (described below) over two levels of physical effort costs. The paradigm was presented as a video game in which participants fought aliens using “blaster” weapons. The blasters were “charged” manually by quick, repeated key presses (physical effort requirement), represented in real-time by an on-screen “charge bar,” which incrementally filled in red with each key press. Blasters only “fired” at an alien if they were fully charged in a pre-allotted amount of time. See **Figure 1** for a trial schematic. (The task is further detailed in the Supplementary Materials.)

### Choice and Effort Conditions

**FIGURE 2:**
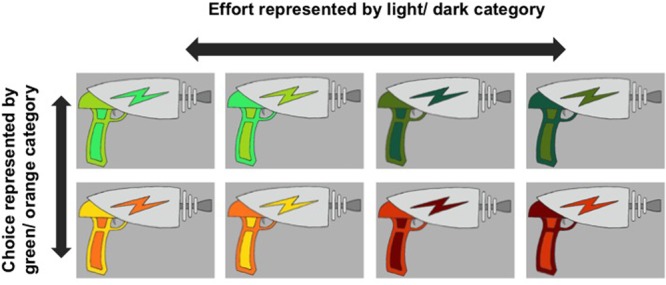
**Example blaster stimuli set.** Across all experiments, conditions were counterbalanced with respect to color (to create four blaster sets per experiment) within the following constraints: color-hue (green vs. orange) represented choice conditions and color-value (lightness vs. darkness) represented effort conditions.

Four variations on this task were implemented, comprising four separate experiments with non-overlapping participant samples. While each individual experiment involved a different subset of conditions (indexed in **Table 1**), the full set of choice (control) and effort (cost) conditions are defined here. The full set of conditions included two levels of effort costs (Low-Requirement and High-Requirement, as defined by the number of key presses required) and three levels of control across choice conditions (Free-Choice, Restricted-Choice, and No-Choice, as defined by the level of control over effort costs conferred by the blaster options offered). The three choice conditions directly manipulated level of control over effort requirements by offering real control, illusory control, and no control, respectively (for further detail see Supplementary Materials). Subtle blaster color categories represented the choice and effort conditions, with two similarly colored exemplar blasters in each category (see **Figure 2**). Participants were given no explicit information regarding how the blaster cue color categories mapped onto choice and effort contingencies. Across all experiments, choice and effort conditions were presented in random order. To fill the charge bar, Low-Requirement blasters required a random number of presses between 11 and 20, while High-Requirement blasters required a random number of presses between 21 and 30. Effort requirements (number of presses) were randomly drawn from ranges that were only subtly different between conditions so that effort contingencies would be somewhat uncertain. This feature of our design allowed implementation of our Restricted-Choice condition (detailed in Experiment 2). See the Supplementary Materials for further detail regarding conditions and stimuli sets.

**Table 1:** Conditions in each experiment.

		Effort (Cost) Conditions	Choice (Control) Conditions
Experiment 1	Effort Requirement Varried, No Choice	Low-Requirement, High-Requirement	No-Choice
Experiment 2	Choice Varried, Low Effort Requirement	Low-Requirement	No-Choice, Controlled-Choice
Experiment 3	Variable Choice and Effort without Free Choice	Low-Requirement, High-Requirement	No-Choice, Controlled-Choice
Experiment 4	Variable Choice and Effort with Free Choice	Low-Requirement, High-Requirement	No-Choice, Controlled-Choice, Free-Choice

### Preference and Performance

Four experiments tested the effects of different combinations of choice (control) and effort (costs) conditions (see **Table 1**) on preference for and performance on choice- and effort- related trials. In order to measure preference for blasters linked to different conditions, participants rated each blaster in the set on seven point Likert-type scale, with a one indicating “I don’t like it at all” and a seven indicating “I like it a lot”. Each blaster was rated before and after playing the game. During both rating sessions all blasters were visible on the screen. As such, ratings inherently represented preference in relation to the whole set of blaster cues for a given game. In each experiment, pre-game ratings were statistically compared to ensure there was no systematic bias in preference before the game. To control for individuals’ prior color predilections, preference was operationalized as an individual’s change in preference (computed by subtracting post- from pre-game ratings). Preference change scores were statistically compared across experimental conditions. Additionally, to determine whether preference changes were different than no change, preference change scores were statistically compared to zero. Performance was operationalized as the percentage of successful trials in a given condition. Thus, the two dependent variables across all four experiments were the change in preference for blasters associated with each condition and the percentage of successful trials in each condition. All error bars throughout the manuscript represent standard error of the mean.

### Set of Experiments

The choice and effort conditions were introduced into the game incrementally across successive experiments so that individual and potential interactive effects of differing levels of cost and control could be detected. Experiment 1 tested the effects of a variable effort requirement alone, using only No-Choice trials of Low- or High-Requirement. Experiment 2 tested the effects of mere choice alone, using only Low-Requirement trials preceded by either No-Choice or Restricted-Choice. Experiments 3 and 4 tested the combined effects of choice and effort, both using two levels of effort (Low-Requirement, High-Requirement) and varied levels of control over effort costs. The key difference between Experiments 3 and 4 is that Experiment 3 utilized two levels of control across choice conditions (Restricted-Choice, No-Choice), while Experiment 4 utilized three levels of control across choice conditions (Free-Choice, Restricted-Choice, No-Choice).

## Experiment 1: Variable Effort Requirement, Constant No-Choice Game

Experiment 1 used trials of either Low- or High-Requirement, while holding the control factor constant with only No-Choice trials in order to test the effects of different levels of effort costs on preference and performance. In this experiment we further sought to establish baseline preference and performance levels for Low- and High-Requirement trials in the absence of choice. The No-Choice condition was implemented by offering participants a single blaster per trial. In line with our first hypothesis, we predicted that participants would show an increased preference for and enhanced performance on Low- compared to High-Requirement trials.

### Experiment 1: Method

The final sample, excluding one subject for whom no data was collected, totaled 36 participants (16 females; mean age = 22.1 years, *SD* = 7.33). Participants were randomly assigned to one of four counterbalanced sets of stimuli (see **Figure 2**). Two stimuli sets were composed of only the green blasters and two sets of only orange. Within all four sets of blaster stimuli, the color-value (lightness vs. darkness) distinguished the levels of effort (Low-Requirement, High-Requirement) in a counterbalanced fashion, with two exemplar blasters in each effort condition. In a single block there were 16 No-Choice trials: eight Low-Requirement and eight High-Requirement. Blocks repeated six times across the game, resulting in a total of 48 No-Choice, Low-Requirement trials and 48 No-Choice, High-Requirement trials.

### Experiment 1: Results

#### Preference

**FIGURE 3:**
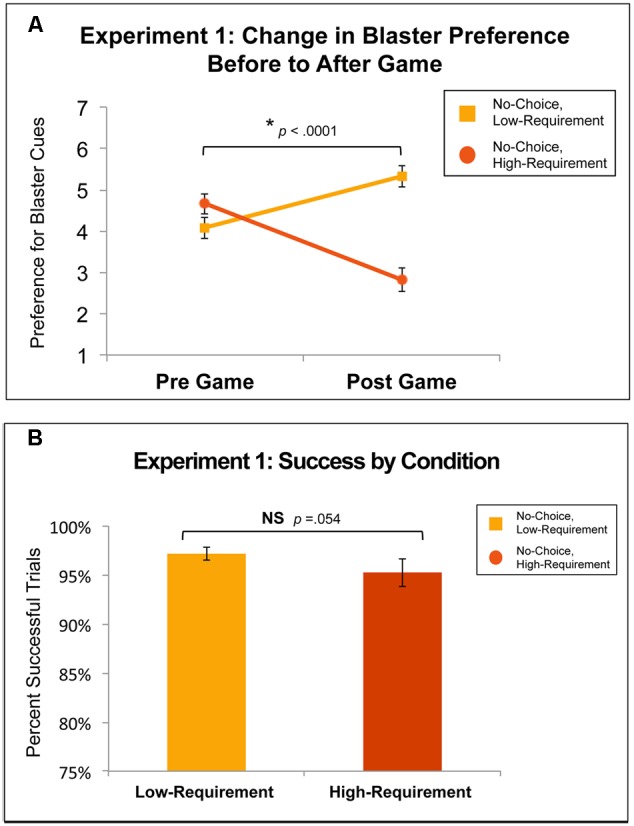
**(A)** Change in preference for conditions in Experiment 1: the Variable Effort Requirement, Constant No-Choice Game. There was a substantial and significant change in preference for Low- compared to High-Requirement blasters (*p* < 0.0001, *d* = 1.47). **(B)** Success rates for conditions in Experiment 1. The difference in success rates between Low- and High-Requirement blasters was moderate but did not reach significance (*p* = 0.054, *d* = 0.29).

**Figure 3A** shows the change in preference for Low- and High-Requirement blaster cues, on a seven point Likert-type scale, from before to after participants played the Experiment 1 Game. Pre-game preference ratings for Low- and High-Requirement blasters were not significantly different [*t*(35) = -1.43, *p* = 0.162]. Preference ratings for Low-Requirement blasters increased from an average pre-game rating of 4.08 (*SD* = 1.55) to an average post-game rating of 5.32 (*SD* = 1.51). For High-Requirement blasters, preference ratings decreased from an average pre-game rating of 4.67 (*SD* = 1.49) to an average post-game rating of 2.82 (*SD* = 1.67). For both Low- and High-Requirement conditions, the change in preference was significantly different from zero (both *p’s* < 0.005). Additionally, the change in preference for Low- compared to High-Requirement blasters differed significantly [*t*(35) = 5.09, *p* < 0.0001, *d* = 1.47], suggesting that the difference between Low- and High- effort requirements was sufficiently learned. There were no gender differences in the change in preference for either condition.

#### Performance

**Figure 3B** shows success rates for Low- and High-Requirement trials in Experiment 1. While success rates were high for both conditions, success for Low-Requirement trials (*M* = 97.1%, *SD* = 4.0%) was higher than for High-Requirement trials (*M* = 95.2%, *SD* = 8.4%), although this moderate difference did not reach significance [*t*(35) = 1.99, *p* = 0.054, *d* = 0.29]. Males had significantly better performance than females for both Low-Requirement [Male: *M* = 98.4%, *SD* = 2.5%; Female: *M* = 95.6%, *SD* = 5%; *t*(34) = 2.24. *p* = 0.031, *d* = 0.71] and High-Requirement [Male: *M* = 98.2%, *SD* = 3%; Female: *M* = 91.5%, *SD* = 11%; *t*(34) = 2.57, *p* = 0.015, *d* = 0.83].

### Experiment 1: Discussion

In this experiment in which only effort costs were varied, we sought to establish baseline levels of preference for and performance on Low-Requirement and High-Requirement conditions when there was no control over effort costs. Based on effort discounting theory, which holds that the value of an outcome is discounted by its associated effort requirement, we hypothesized that participants would show enhanced preference and performance for the Low- compared to High-Requirement condition. These hypotheses were supported, as evidenced by strong preference for Low-Requirement blasters. High success rates across conditions confirmed that both the Low and High levels of effort were achievable for subjects. Success rates were moderately higher in the Low-Requirement condition, although this difference did not reach significance. Thus, results from subsequent experiments can be interpreted in light of participants preferring Low- to High-Requirement and showing a modest, albeit non-significant, boost in performance in the Low-Requirement condition. While men and women did not show a difference in their preferences for the effort conditions, men performed significantly better than women across both effort conditions. As the task was disguised a video game, performance differences may have been due to men potentially having more favorable perceptions of the task.

## Experiment 2: Variable Choice, Constant Low Effort Requirement Game

Experiment 2 used Low-Requirement trials of either Restricted- or No-Choice to test the effects of different levels of control (illusory control and no control, respectively) on preference and performance, with effort level held constant. In this experiment we sought to establish baseline preference and performance levels for Restricted- and No-Choice trials in the context of a low effort requirement. While the No-Choice condition offered only one blaster for participants to use, the Restricted-Choice conditions offered two blasters from the same effort requirement category (Low-Requirement in this experiment). Thus, the Restricted-Choice condition did not grant any actual control over effort requirements. However, control is often inferred even when individuals actually possess none (Langer, 1975; Wegener and Wheatley, 1999; Davis et al., 2000; Clark et al., 2009). To further promote this tendency of presuming personal control, three features were implemented including: (1) giving participants no explicit information regarding the choice and effort conditions, (2) subtle mapping of blaster cues colors onto choice and effort contingencies that had to be learned through experience, and (3) drawing effort requirements from a range (in this experiment, the Low-Requirement range of 11–20 presses) so that the effort required was somewhat ambiguous. While these features were implemented across all experimental conditions, we expected that the ambiguity created would particularly facilitate perceptions of control when participants were given a choice (Restricted-Choice condition). Although the control offered by the Restricted-Choice condition was only illusory, even illusory control enhances valuation and intrinsic motivation (Cordova and Lepper, 1996; Clark et al., 2009; Leotti and Delgado, 2011; Murayama et al., 2015). Thus, in line with our second general hypothesis, we predicted that participants would show enhanced preference and performance for Restricted- compared to No-Choice trials, even though the Restricted-Choice condition provided no actual means for reducing effort costs. On the other hand, if participants (accurately) perceived that the Restricted-Choice condition offered no actual control, then we would not expect to see enhancements in motivational outcomes for the Restricted- relative to the No-Choice condition (Moller et al., 2006).

### Experiment 2: Method

Thirty-four participants were recruited and completed the experiment (17 females; mean age = 21.1 years, *SD* = 5.09). Participants were randomly assigned to one of four counterbalanced sets of stimuli (see **Figure 1**). Two stimuli sets were composed of only light colored blasters (light green and light orange) and two sets of only dark colored blasters (dark green and dark orange). Within all four sets, color-hue (green vs. orange) distinguished choice conditions (Restricted-Choice, No-Choice) in a counterbalanced fashion, with two exemplar blasters in each choice condition. The Restricted-Choice condition was implemented by offering a choice between two blasters of slightly different colors that were both from the Low-Requirement condition. Importantly, choice in this condition did not confer any control over effort costs, however, this may not have been apparent to participants. In a single block there were 32 Low-Requirement trials: 16 Restricted-Choice and 16 No-Choice. Blocks repeated three times across the game, resulting in a total of 48 Restricted-Choice, Low-Requirement trials and 48 No-Choice, Low-Requirement trials.

### Experiment 2: Results

#### Preference

**FIGURE 4:**
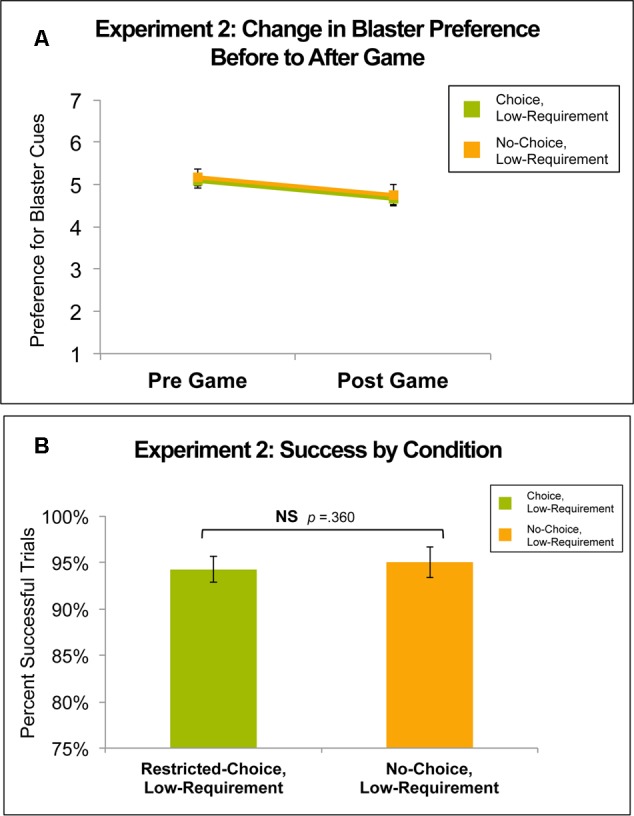
**(A)** Change in preference for conditions in Experiment 2: the Variable Choice, Constant Low-Requirement Game. There was no difference in preference change between Restricted- and No-Choice blasters. **(B)** Success rates for conditions in Experiment 2. There was no difference in success rates between Restricted- and No-Choice blasters (*p* = 0.360, *d* = 0.09).

**Figure 4A** shows the change in preference for Restricted- and No-Choice blaster cues from before to after participants played the Experiment 2 Game. Pre-game preference ratings for Restricted- and No-Choice blasters were not significantly different [*t*(33) = -0.39, *p* = 0.699]. Across both the Restricted- and No-Choice conditions, preference ratings decreased from similar average pre-game ratings of 5.07 (*SD* = 0.92) and 5.16 (*SD* = 1.14) to similar post-game ratings of 4.66 (*SD* = 1.04) and 4.75 (*SD* = 1.38), respectively. For both conditions, the change in preference did not significantly differ from zero [Restricted-Choice: *t*(33) = -1.80, *p* = 0.081; No-Choice: *t*(33) = -1.60, *p* = 0.119] and there was no difference in the pre- to post- change in preference between the Restricted- and No-Choice conditions. From before to after the game, preference for both conditions decreased approximately 0.06 of a point on a seven point Likert-type scale. There were no gender differences in preference, nor in performance for either condition.

#### Performance

**Figure 4B** shows success rates for Restricted- and No-Choice trials in Experiment 2. Participants performed similarly in the two conditions, successfully completing 95% (*SD* = 7.9%) of No-Choice trials and 94.2% (*SD* = 9.9%) of Restricted-Choice trials [*t*(33) = 0.93, *p* = 0.360, *d* = 0.09].

### Experiment 2: Discussion

This experiment employed conditions of No-Choice and Restricted-Choice for the purpose of determining the impact of no control and illusory control, respectively, on preference for and performance on trials requiring only low effort levels. As illusory control can have a beneficial effect on valuation and intrinsic motivation (Cordova and Lepper, 1996; Clark et al., 2009; Leotti and Delgado, 2011; Murayama et al., 2015), we hypothesized that participants would show enhanced intrinsic motivation and thus improved motivational outcomes for Restricted- compared to No-Choice trials, in line with our second general hypothesis. However, this hypothesis was not supported, as there were no meaningful or significant differences between the two choice conditions. Across both choice conditions, preference for blasters decreased very slightly and to the same degree. These changes in preference were neither significantly different from one another nor significantly different from no change. Similarly, there was no difference in success rates between the two conditions. Thus, the provision of illusory control appeared to have no substantial impact on participants’ preference or performance in this experiment. While previous studies have found positive motivational effects of choices offering only illusory control, many such studies involved choices in a context of rewarding outcomes or intrinsically motivating situations. However, there is evidence that a context of costs may reduce the positive effects of choice (Iyengar and Lepper, 2000; Gourville and Soman, 2005; Fleming et al., 2010). For example, a series of experiments by Leotti and Delgado (2014) demonstrated that losses, compared to gains, can diminish the affective experience of exercising personal control via making choices. As effort is weighed as a cost in the decision making process in much the same manner as monetary losses (Botvinick et al., 2009; Kurniawan et al., 2010), it is possible that in the context of the present task, effort costs overshadowed the potential motivational benefits of the Restricted-Choice condition. Our results are consistent with work by Moller et al. (2006) who also examined choice in the context of effortful tasks and found no performance benefits from choices that limited the expression of personal control. Thus, the results from the current experiment suggest that when choice is limited and offers only illusory control, an effortful context may undermine the potential motivational benefits often associated with choice.

## Experiment 3: Variable Choice and Effort, Without Free Choice Game

The Variable Choice and Effort, Without Free Choice Game used Restricted- and No-Choice trials of both Low- and High-Requirement to test the combined influence of different levels of control and cost factors on preference and performance in a 2x2 design. Within this experiment we examined hypotheses two and three: that preference and performance ratings would favor conditions where greater control is perceived, and that low control but high effort would have the combined influence of diminishing motivational outcomes. As in Experiment 2, the Restricted-Choice condition did not confer any control over effort costs, as the blaster options offered in Restricted-Choice were always within the same effort category (e.g., a choice between two High-Requirement blasters). Thus, the perception of control was free to subjectively vary, while actual control was effectively zero across both choice conditions. Although there was no effect of choice on preference or performance in Experiment 2, which involved only low effort requirements, we predicted that the addition of a High-Requirement condition might elicit a positive motivational effect from the Restricted-Choice condition. We specifically hypothesized that the contrast of having two levels of effort might increase the salience of personal control, as participants tried to exercise control to avoid the higher effort costs (in line with participant preference for Low-Requirement established in Experiment 1). As with all of the experiments, the choice and effort contingencies were obscured such that actual levels of control over effort costs and how these contingencies mapped onto blaster cues were somewhat ambiguous. For example, effort cost requirements (number of presses) for the two conditions were drawn from ranges that were consecutive to one another (Low-Requirement 11–20 presses; High-Requirement 21–30 presses), thus making effort costs somewhat difficult to characterize. Such ambiguities in the task left a margin of uncertainty for participants to make inferences regarding the degree to which Restricted-Choice afforded control over effort costs. We predicted that participants would infer control in the Restricted-Choice condition, which would boost intrinsic motivation for effort trials in this condition. Thus, in line with our second general hypothesis, we predicted participants would show enhanced preference and performance for Restricted-Choice compared to No-Choice trials. In-line with hypothesis three, we also specifically anticipated that the additional effort requirements of the High-Requirement condition might undermine motivation, especially as high effort costs intersected with low levels of personal control. Thus, we predicted that the most severe decrements in motivational outcomes would be observed in the No-Choice, High-Requirement condition.

### Experiment 3: Method

Thirty-three participants completed the experiment (13 females, 2 other gender; mean age = 21.7 years, *SD* = 4.83). Participants were randomly assigned to one of four counterbalanced sets of stimuli. Color-hue category (green vs. orange) distinguished levels of choice (Restricted-Choice, No-Choice) and color-value category (lightness vs. darkness) distinguished levels of effort (Low-Requirement, High-Requirement); within these constraints, conditions were fully counterbalanced across color categories, creating four sets of blaster stimuli with two exemplar blasters in each of 2x2 conditions (see **Figure 2**). Across a single block there were 16 Restricted-Choice trials (eight Low-Requirement and eight High-Requirement) and 16 No-Choice trials (eight Low-Requirement and eight High-Requirement). Blocks repeated four times across the game resulting in a total of 32 Restricted-Choice, Low-Requirement trials; 32 Restricted-Choice, High-Requirement trials; 32 No-Choice, Low-Requirement trials; and 32 No-Choice, High-Requirement trials.

### Experiment 3: Results

#### Preference

**FIGURE 5:**
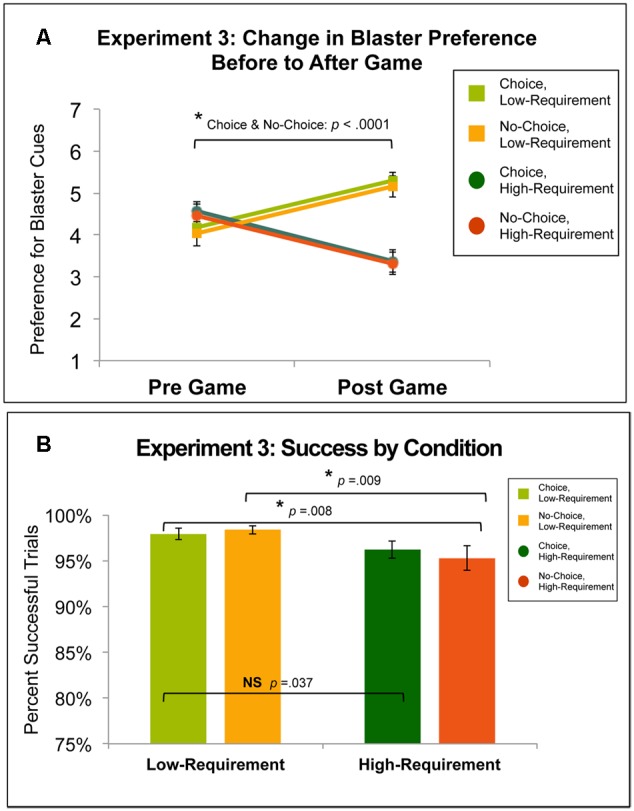
**(A)** Change in preference for conditions in Experiment 3: the Variable Choice and Effort Without Free Choice Game. The increase in preference for Low- blasters was substantially and significantly different than the decrease in preference for High-Requirement blasters for both Restricted- and No-Choice (both *p*’s < 0.0001, both *d*’s > 1). **(B)** Success rates for conditions in Experiment 3. There was a main effect of effort (*p* = 0.003), such that success rates for Low- blasters were significantly greater than success rates for High-Requirement blasters for No-Choice (*p* = 0.009, *d* = 0.54) and this difference approached the Bonferroni-corrected significance threshold for Restricted-Choice (*p* = 0.037, *d* = 0.38). Additionally, success rates in No-Choice, High-Requirement were significantly lower than in Restricted-Choice, Low-Requirement (*p* = 0.008, *d* = –0.44).

**Figure 5A** shows the change in preference for all choice and effort conditions from before to after participants played the Variable Choice and Effort Without Free Choice Game. Bonferroni-corrected pairwise *t*-test of pre-game preference ratings did not reveal any significant differences among the four conditions. Across choice conditions, preference for Low-Requirement increased from pre-game (Restricted-Choice, Low: *M* = 4.18, *SD* = 1.27; No-Choice, Low: *M* = 4.03, *SD* = 1.66) to post-game (Restricted-Choice, Low: *M* = 5.29, *SD* = 1.22; No-Choice Low: *M* = 5.15, *SD* = 1.47) and preference for High-Requirement blasters decreased from pre-game (Restricted-Choice, High: *M* = 4.56, *SD* = 1.42; No-Choice, High: *M* = 4.45, *SD* = 1.7) to post-game (Restricted-Choice, High: *M* = 3.38, *SD* = 1.54; No-Choice, High: *M* = 3.32, *SD* = 1.49). Across all conditions, changes in preference were significantly different from zero (all *p’s* < 0.005). A 2x2 ANOVA of the change in preference data, indicated a main effect of effort [*F*(1,32) = 37, *p* < 0.0001], such that the increase in preference for Low-Requirement blasters was significantly different (using the Bonferroni-corrected significance threshold of *p* = 0.025) than the decrease in preference for High-Requirement blasters for both the Restricted-Choice [*t*(32) = 4.82, *p* < 0.0001, *d* = 1.32] and No-Choice [*t*(32) = 4.92, *p* < 0.0001, *d* = 1.21] conditions. However, there was no main effect of choice [*F*(1,32) = 0.022, *p* = 0.882], as the Restricted-Choice and No-Choice conditions (collapsing across effort conditions) showed virtually no difference in this experiment [*t*(32) = -0.15, *p* = 0.882]. There was no interaction of the choice and effort conditions in the before to after game preference change [*F*(1,32) = 0.003, *p* = 0.957]. When gender was added as a between subjects factor in the ANOVA, there were no significant effects related to gender for either the preference or performance measures.

#### Performance

**Figure 5B** shows success rates for all conditions in the Variable Choice and Effort Without Free Choice Game. Success rates were high (above 95%) for all four conditions (Restricted-Choice, Low-Requirement: *M* = 97.9%, *SD* = 3.4%; Restricted-Choice, High-Requirement: *M* = 96.2%, *SD* = 5.3%; No-Choice, Low-Requirement: *M* = 98.4%, *SD* = 2.6%; No-Choice, High-Requirement: *M* = 95.3%, *SD* = 7.7%). A 2x2 ANOVA indicated a main effect of effort [*F*(1,32) = 10.679, *p* = 0.003], such that success rates for Low-Requirement blasters were significantly greater than success rates for High-Requirement blasters for the No-Choice condition [*t*(32) = 2.77, *p* = 0.009, *d* = 0.54] and this difference for the Restricted-Choice condition approached the Bonferroni-corrected significance threshold of *p* = 0.025 [*t*(32) = 2.18, *p* = 0.037, *d* = 0.38]. However, there was no main effect of choice [*F*(1,32) = 0.181, *p* = 0.674], as the Restricted-Choice and No-Choice conditions (collapsing across effort conditions) showed virtually no difference [*t*(32) = 0.425, *p* = 0.674]. There was no interaction of the choice and effort conditions [*F*(1,32) = 1.267, *p* = 0.269].

As we had an *a priori* hypothesis that the greatest motivational decrements would be observed in the No-Choice, High-Requirement condition, we also examined performance differences in this condition relative to the other conditions. While the decrement in performance in the No-Choice, High-Requirement condition was significant when compared to both No-Choice, Low-Requirement (as already stated) and Restricted-Choice, Low-Requirement [*t*(32) = -2.81, *p* = 0.008, *d* = -0.44], the decrement was not significant in comparison to Restricted-Choice, High-Requirement [*t*(32) = -0.88, *p* = 0.385].

### Experiment 3: Discussion

This experiment employed conditions of No-Choice and Restricted-Choice across both Low-Requirement and High-Requirement trials to determine the combined impact of varying both costs and control over effort requirements on motivational outcomes in a 2x2 design. While the Restricted-Choice condition did not confer any actual control over effort costs, experimental contingencies were ambiguous so that participants might infer control (though illusory), and thus, experience enhanced motivation in this condition. We hypothesized that the varied effort requirements might create a direct contrast effect, making the choice condition more salient in Experiment 3 than in Experiment 2. Thus, in line with our second general hypothesis, we anticipated increased intrinsic motivation when participants were given a choice and, therefore, enhanced motivational outcomes for the Restricted- compared to No-Choice condition. However, this hypothesis was not supported. Only the effort, but not the choice condition had an effect on preference and performance. Replicating results from Experiment 1 and supporting our first general hypothesis, participants in Experiment 3 both preferred and were more successful on Low-Requirement compared to High-Requirement trials. Replicating results from Experiment 2, Restricted-Choice compared to No-Choice did not significantly affect preference or performance. Thus across Experiments 2 and 3, there was no evidence that the mere provision of choice, conferring only illusory control, had any impact on participants’ preference or performance. These findings are in line with studies demonstrating that mere choice, devoid of opportunities for personal control, does not enhance motivation (Reeve et al., 2003; Moller et al., 2006). Further, these results suggest participants may have experienced the Restricted-Choice condition, which offered options that were only superficially different, as limiting their opportunity to express control. Evidence suggests when individuals experience conditions as *controlling* their behavior rather than providing them with an opportunity for control, intrinsic motivation is undermined (Pittman et al., 1980; Deci and Ryan, 1987; Patall et al., 2008).

While we did not observe a differential effect between the choice conditions related to increasing effort requirements, it is possible that both effort conditions required an effort cost beyond some threshold at which the potential motivational benefits of choice are undermined–particularly when choices offer no real control. Thus, the null effect of choice in this experiment may be due to both low levels of personal control (in both the Restricted- and No-Choice conditions) and due to effort costs (across both effort conditions).

While our second general hypothesis was not supported, performance results are consistent with our third general hypothesis, which posited that the greatest motivational deficits would occur when the lowest levels of personal control met the highest effort costs. Participants were least successful in the No-Choice, High-Requirement condition, suggesting a lack of control and effort costs may exert a conjoint influence to undermine motivation. To further parse the effects of personal control and effort costs on the motivational consequences of choice, future studies should examine the effects of differing levels of control across a greater range of effort costs, including a no-effort condition. Nonetheless, results from the current study suggest that in the context of effort requirements, a choice conferring only illusory control may not sufficiently bolster perceptions of personal control or override decision-related costs to enhance motivation.

## Experiment 4: Variable Choice and Effort, With Free Choice Game

The Variable Choice and Effort, With Free Choice Game was similar to Experiment 3, but introduced a new choice condition: Free-Choice. In the Free-Choice condition, participants were given a choice between one Low- and one High-Requirement blaster and were allowed to freely choose which they preferred to use. Thus, Experiment 4 used Free-, Restricted- and No-Choice trials of both Low- and High-Requirement to test the combined influence of different levels of personal control and effort costs on preference and performance. As before, the Restricted-Choice condition did not offer any actual control over effort requirements; however, the Free-Choice condition did. We sought to determine whether the provision of Free-Choice would increase perceptions of control to enhance intrinsic motivation for freely chosen effort trials. In line with our second general hypothesis, we generally predicted that motivational outcomes would be enhanced correspondent to the level of control conferred by choice (Free-Choice > Restricted-Choice > No-Choice). However, choice conditions could not be fully dissociated for the preference data, because the Free- and Restricted-Choice conditions utilized the same set of blaster cues (and preference was calculated via pre- and post- game ratings of the cues). Thus, preference could only be determined for the collapsed Choice condition (encompassing both Free- and Restricted-Choice). Therefore, for preference data, we hypothesized that Choice trials would be preferred over No-Choice trials. Furthermore, as the only difference between the two conditions comprising the collapsed Choice condition was whether the blasters options presented were from the same (Restricted-) or different (Free-) effort categories, we further anticipated that perceptions of control elicited by Free-Choice trials would generalize to the Restricted-Choice trials as well. Therefore, for the performance data, we hypothesized that we would observe enhanced performance in both Free- and Restricted-Choice conditions. In line with our third general hypothesis, we also predicted that any observed deficits in motivational outcomes would occur at the intersection of the lowest levels of personal control (only No-Choice in this experiment) and the highest effort costs (High-Requirement).

### Experiment 4: Method

The final sample, with one participant excluded for not following directions, totaled 33 participants (22 females; mean age = 21.7 years, *SD* = 4.21). Participants were randomly assigned to one of four counterbalanced sets of stimuli. As in Experiment 3, conditions were fully counterbalanced across color categories, creating four sets of blaster stimuli (see **Figure 2**). In terms of the blaster stimuli, this Experiment only differed from Experiment 3 in that the color-hue category (green vs. orange) representing Choice, represented both the Free- and Restricted-Choice conditions together. Across a single block there were 16 No-Choice trials (eight Low-Requirement and eight High-Requirement) and 16 Choice trials. Within the Choice trials, there were eight Restricted-Choice trials (four Low-Requirement and four High-Requirement) and eight Free-Choice trials (in which participants could freely choose Low- or High-Requirement). Blocks repeated four times across the game resulting in a total of 32 No-Choice, Low-Requirement trials, 32 No-Choice, High-Requirement trials, at least 16 Choice, Low-Requirement and at least 16 Choice, High-Requirement trials, plus an additional 32 Free-Choice trials (with effort level dependent on participants’ choices).

### Experiment 4: Results

#### Preference

**FIGURE 6:**
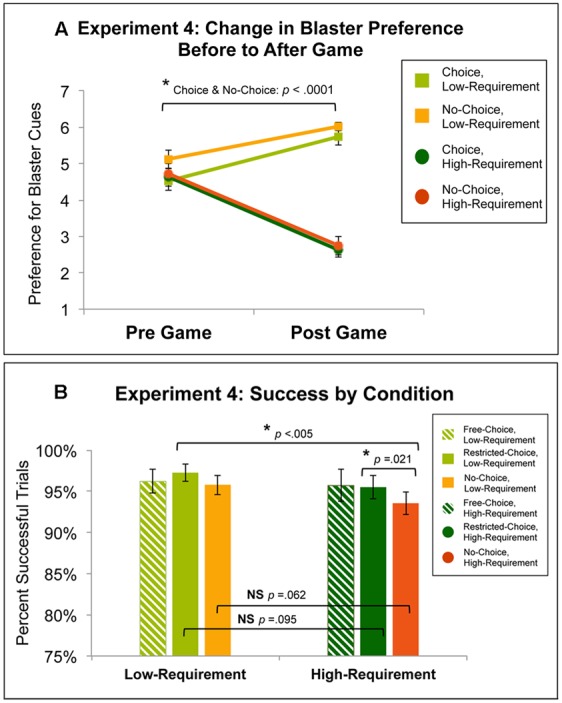
**(A)** Change in preference for conditions in Experiment 4: the Variable Choice and Effort With Free Choice Game. There was a main effect of effort (*p* < 0.0001), such that the increase in preference for Low- blasters was substantially and significantly different than the decrease in preference for High-Requirement blasters for both Choice and No-Choice (both *p*’s < 0.0001, both *d*’s > 1). **(B)** Success rates for conditions in Experiment 4. A 2x2 ANOVA excluding the Free-Choice condition, produced a main effect of effort (*p* = 0.025), although the pairwise comparisons across effort requirement conditions did not reach significance for either Choice (*p* = 0.095, *d* = 0.26) or No-Choice (*p* = 0.062, *d* = 0.30). There was also a main effect of choice (*p* = 0.015), such that success rates for Choice were significantly greater than success rates for No-Choice in the High-Requirement (*p* = 0.021, *d* = 0.24), but this difference did not reach significance for the Low-Requirement (*p* = 0.187, *d* = 0.24). Also, success rates for No-Choice, High-Requirement were significantly different than in Restricted Choice, Low-Requirement (*p* < 0.005, *d* = –0.54).

**Figure 6A** shows the change in preference for all choice and effort conditions from before to after participants played the Variable Choice and Effort With Free Choice Game. Bonferroni-corrected pairwise *t*-test of pre-game preference ratings did not reveal any significant differences among the four conditions. Across both choice conditions, preference for Low-Requirement blasters increased from pre-game (Choice, Low: *M* = 4.52, *SD* = 1.45; No-Choice, Low: *M* = 5.13, *SD* = 1.43) to post-game (Choice, Low: *M* = 5.73, *SD* = 1.31; No-Choice, Low: *M* = 6.02, *SD* = 0.68) and preference for High-Requirement blasters decreased from pre-game (Choice, High: *M* = 4.63, *SD* = 1.41; No-Choice, High: *M* = 4.72, *SD* = 1.63) to post-game (Choice, High: *M* = 2.63, *SD* = 1.12; No-Choice, High: *M* = 2.75, *SD* = 1.45). Across all conditions, changes in preference were significantly different from zero (all *p’s* < 0.005). A 2x2 ANOVA of the change in preference data, indicated a main effect of effort [*F*(1,31) = 46.98, *p* < 0.0001], such that the increase in preference for Low-Requirement blasters was significantly different (using the Bonferroni-corrected significance threshold of *p* = 0.025) than the decrease in preference for High-Requirement blasters for both Choice [*t*(31) = 6.87, *p* < 0.0001, *d* = 1.86] and No-Choice [*t*(31) = 5.06, *p* < 0.0001, *d* = 1.43]. However, there was no main effect of the choice [*F*(1,31) = 0.30, *p* = 0.59] and no interaction of the choice and effort conditions in the preference change [*F*(1,31) = 0.44, *p* = 0.511]. When gender was added as a between subjects factor in the ANOVA, there were no significant effects related to gender for either the preference or performance measures.

#### Performance

**Figure 6B** shows success rates for all conditions in the Variable Choice and Effort With Free Choice Game. Success rates were high (above 93%) across all six conditions (Free-Choice, Low-Requirement: *M* = 96.2%, *SD* = 8.3%; Free-Choice, High-Requirement: *M* = 95.7%, *SD* = 11.1%; Restricted-Choice, Low-Requirement: *M* = 97.3%, *SD* = 5.9%; Restricted-Choice, High-Requirement: *M* = 95.5%, *SD* = 8%; No-Choice, Low-Requirement: *M* = 95.8%, *SD* = 6.8%; No-Choice, High-Requirement: *M* = 93.6%, *SD* = 7.7%). The 3x2 ANOVA including all three choice conditions and both effort conditions did not reveal any significant differences among the conditions [*F*(1,31) = 0.233, *p* = 0.793].

We also decided to analyze the performance data in a 2x2 fashion, with Free-Choice trials excluded. This analysis was performed for several reasons: (1) we wanted to perform an analysis comparable to that in Experiment 3; (2) treating Free-Choice and Restricted-Choice as separate conditions may not have accurately reflected participants’ experience of the game as both types of choice were implemented utilizing the same set of blaster cues and thus may have been indistinguishable to participants; (3) collapsing Free- and Restricted-Choice into a single Choice condition was not appropriate as the Free-Choice condition involved a higher percentage of Low-Requirement trials than the two other choice conditions (in which Low- and High-Requirement were balanced); and (4) average success scores were computed from fewer data points in the Free-Choice, High-Requirement condition as participants tended not to choose High- blasters when given a Free-Choice and three subjects never chose a High- blaster during a Free-Choice trial. Thus, performance was analyzed in a 2x2 fashion, excluding Free-Choice trials. While this analysis paralleled the one performed in Experiment 3, the key difference here is that the Free-Choice condition may have still exerted an influence on the context in which participants experienced Restricted-Choice in Experiment 4.

The 2x2 ANOVA with factors of Restricted- and No-Choice and Low- and High- Requirement revealed a main effect of effort [*F*(1,31) = 5.57, *p* = 0.025]. While success rates for Low-Requirement blasters were greater than success rates for High-Requirement blasters, *post hoc* pairwise comparisons did not reach significance for either of the Choice [*t*(31) = 1.72, *p* = 0.095, *d* = 0.26] or the No-Choice [*t*(31) = 1.94, *p* = 0.062, *d* = 0.30] conditions.

Further, the 2x2 ANOVA revealed a main effect of choice [*F*(1,31) = 6.64, *p* = 0.015], such that success rates for the Choice condition were significantly greater than success rates for the No-Choice condition for High-Requirement [*t*(31) = 2.43, *p* = 0.021, *d* = 0.24], while this difference was not significant for Low-Requirement [*t*(31) = 1.35, *p* = 0.187, *d* = 0.24]. There was no interaction of the choice and effort factors [*F*(1,31) = 0.13, *p* = 0.725].

As we had an *a priori* hypothesis that the greatest motivational decrements would be observed in the No-Choice, High-Requirement condition, we also examined performance differences in this condition relative to other conditions. While the decrement in performance in No-Choice, High-Requirement was significant when compared to Choice, High-Requirement (as already stated) and Choice, Low-Requirement [*t*(31) = -4.27, *p* < 0.005, *d* = -0.54], this decrement in performance did not reach the Bonferroni-corrected significance threshold of *p* = 0.017 when compared to No-Choice, Low-Requirement [*t*(31) = -1.94, *p* = 0.062, *d* = -0.30].

### Experiment 4: Discussion

This experiment employed conditions of No-Choice, Restricted-Choice, and Free-Choice across both Low-Requirement and High-Requirement trials to determine the combined impact of varying both costs and control on motivational outcomes. The design of Experiment 4 was similar to Experiment 3, except that only half of the Choice trials were Restricted-Choice trials (split evenly between Low- and High-Requirement), and the other half of the Choice trials were Free-Choice. Free-Choice trials offered participants a choice between a High- and a Low-Requirement blaster and they freely chose which they preferred to use. Given the Restricted-Choice condition in Experiments 2 and 3 did not enhance preference or performance, we sought to determine whether the provision of Free-Choice, a choice that did actually confer control over effort costs, would enhance intrinsic motivation. In line with our second general hypothesis, we predicted that outcomes would be enhanced correspondent to the level of control conferred. While preference data did not support this hypothesis, it did replicate the findings from Experiments 1–3, with an increase in preference for Low-Requirement cues, a decrease for High-Requirement cues, and no apparent effect of Choice (representing both Free- and Restricted-Choice) on preference.

Performance data, however, did demonstrate main effects of not only effort but also choice. While the 3x2 analysis of success rates did not yield significant effects, this may have been due to the two choice conditions being indistinguishable to participants as they were both represented by the same blaster cues. Thus, we also analyzed performance data in a 2x2 fashion, excluding Free-Choice trials, in order to have an analysis comparable to that in Experiment 3 and to remove the low effort advantage conferred by Free-Choice. Results from this analysis replicated results from Experiments 1 and 3, and supported our first hypothesis, with participants performing significantly better on Low- than High-Requirement trials. Interestingly, while effort effect sizes in Experiment 3 (without Free-Choice) were moderate to large, effort effect sizes in this experiment (that did include Free-Choice) were small to moderate, suggesting that in the context of Free-Choice, the magnitude of effort’s effect was reduced.

Importantly, results from this analysis also supported our second hypothesis that intrinsic motivation and thus motivational outcomes would be enhanced correspondent to the level of control conferred by the choice condition. Experiment 4 was the only experiment to offer actual control over effort costs and the only experiment in which participants performed better on effortful trials in which they were given a choice compared to those in which they were not. Interestingly, the choice effect sizes for performance in this experiment were comparable to the effort effect sizes. This suggests that in the context of Free-Choice, effort and choice may have a similar effect magnitude on performance. This positive motivational effect of choice was present even when Free-Choice trials were excluded. Thus, even when the lower effort advantage conferred by Free-Choices was removed from the analysis, the motivational effect of personal control, uniquely offered in this experiment, still had a positive impact on the illusory control condition, a previously unmotivating condition.

Thus, in contrast to Experiments 2 and 3, in which Restricted-Choices were offered alone and there was no positive effect of choice, the results of Experiment 4 suggest that the provision of occasional free choices may be sufficient to provide motivational benefits in an effortful, and otherwise uncontrollable context. While participants were not more successful on Free-Choice trials than on Restricted-Choice trials, this may have been due to both the Restricted- and the Free-Choice trials utilizing the same set of blaster stimuli. Given the subtle differences between these two Choice conditions, it is possible that participants may not have explicitly distinguished between Free- and Restricted-Choice conditions, and rather, perceived all the choices as conferring some control over effort costs.

In line with our third hypothesis, and consistent with results from Experiment 3, we again observed the lowest levels of performance in the No-Choice, High-Requirement condition, again supporting the notion that low levels of personal control and high levels of costs may combine to undermine motivation (Iyengar and Lepper, 2000; Reeve et al., 2003; Moller et al., 2006; Fleming et al., 2010; Patall, 2012). While choosing did significantly enhance performance, preference remained unaffected by the provision of choice. It is possible that despite perceptions of control positively affecting performance on immediately subsequent trials, this apparent motivational effect was not integrated across trials to create a stronger preference for cues associated with choice. It is also possible that effort costs undermined the preference for having a choice but not choice-linked performance. Future research might investigate possible dissociable effects of perceptions of control on performance and preference and how these effects may dynamically interact with one another.

## General Discussion

The present research attempted to disambiguate how control and cost factors affect the motivational consequences of choosing, across differing levels of personal control, within an effortful task. Across four experiments we tested the hypotheses that intrinsic motivation and thus motivational outcomes would be: (I) enhanced when lower compared to higher effort was required, (II) enhanced correspondent to the level of control conferred by choice, and (III) diminished when the lowest levels of personal control intersected with the highest effort requirements. These hypotheses were generally, although not always specifically supported.

Our first hypothesis was premised upon effort discounting theory, which holds that effort decreases the utility of related outcomes (Botvinick et al., 2009; Kurniawan et al., 2013). Thus, we hypothesized that participants would both prefer and perform better on lower effort requirements. This hypothesis was supported for both preference and performance across all experiments that varied effort (1, 3, 4). This result held despite the time allowance for effort trials being calibrated so that both levels of effort were achievable.

Our second hypothesis, that intrinsic motivation and thus motivational outcomes would be enhanced according to the level of control offered by the choice condition was generally supported, although the threshold for an effect of control was not what was predicted. As much research has demonstrated the motivational benefits of personal control and even of illusory control (Langer, 1975; Langer and Rodin, 1976; Alloy and Abramson, 1979; Ryan and Deci, 2000; Leotti et al., 2010, 2015), we predicted that our illusory control (Restricted-Choice) condition would enhance motivational outcomes. However, our results indicated that illusory control alone may not enhance intrinsic motivation in a context of effortful exertion. In Experiments 2 and 3, which only offered illusory control, no beneficial effects of choosing were observed. However, in the only experiment to offer real control over effort costs (Experiment 4), there was a positive effect of choice on performance. Importantly, choice effect sizes in this experiment were comparable to effort effect sizes, suggesting that in the context of real control, choice and effort had similar magnitude effects on intrinsic motivation to perform. Together, these results suggest that while real control enhances intrinsic motivation in the face of effort costs, illusory control alone may not be sufficient.

Based on evidence that low levels of personal control and heightened costs associated with a decision may combine to produce a particularly damaging coalition, our third hypothesis predicted that we would observe the largest decrements in motivation when personal control was at its lowest and cost was at its highest. This hypothesis was largely confirmed in both of the experiments testing control and cost together (Experiments 3 and 4), suggesting that the combined effect of low personal control and a context of high costs can lead to diminished intrinsic motivation.

Another interesting effect to emerge was that, while illusory control alone did not enhance intrinsic motivation, the positive effects of free choice appeared to generalize from the real control condition to the illusory control condition. That is, when real control was offered, there was enhanced performance in illusory control trials, even when the direct influence of Free-Choice trials (and the low-effort advantage conferred by Free-Choice) was removed. This suggests conditions of illusory control can be contextualized by the opportunity for real control, enhancing motivational effects of illusory control when it otherwise might not be associated with increased motivation.

Together the pattern of results across all of our experiments provides support for our overarching hypotheses, suggesting that personal control provides benefits to intrinsic motivation while effort costs undermine these benefits. Further, our results suggest there may be interplay between control and cost factors that may influence the motivational consequences of making choices. For example, our study demonstrated that under conditions of effort costs, low levels of personal control might not override these costs to boost intrinsic motivation in a decision making context. This is consistent with work suggesting there may be a framing effect for decisions such that choice may lose its desirability and advantageous features in the context of loss frames and high costs (Iyengar and Lepper, 2000; Fleming et al., 2010; Leotti and Delgado, 2014). For example, Botti and Iyengar (2004) found beneficial effects of choosing when options were attractive but found detrimental effects of choosing when participants chose from among unattractive options. Similarly, effortful contexts may negate the benefits of mere choice and require higher levels of personal control to furnish motivation and confer value to the decision making process. Our study also demonstrated that deriving positive motivational effects from illusory control, while commonly evidenced (e.g., Cordova and Lepper, 1996; Clark et al., 2009; Murayama et al., 2015), may be a fragile effect, easily unraveled by factors such as effort costs. This interpretation is consistent with prior work demonstrating motivational benefits resulting from choices that confer personal control but not from the mere act of choice (Reeve et al., 2003; Moller et al., 2006).

The present study also had several limitations. First, while we directly manipulated personal control across our choice conditions, no self-report manipulation checks were included and, thus, we can only infer that it was indeed perceptions of personal control that led to the motivational effects observed in Experiment 4. Future work should include self-report measures to ensure that perceptions of personal control match corresponding manipulations of control. Second, in future work, measures of motivation should be refined and potential modulating factors should be tested. For example, while we used preference and performance as measures of motivation across conditions, we did not include a self-report measure of intrinsic motivation for the different choice and effort conditions. Self-reported intrinsic motivation may have more directly addressed study hypotheses. Further, the self-relevance of task goals has been demonstrated to be an important factor in determining motivation and effort exertion (Gendolla and Richter, 2010). While the current work did not examine self-relevance, future work would benefit from the consideration of such closely related theoretical constructs. Third, while evidence that the worst rates of performance occurred under conditions of highest effort and lowest personal control does suggest that effort costs undermine the positive benefits of making choices, a no-effort condition would have allowed us to directly contrast the effects of choice within and without a context of effort. To more directly parse the effects of personal control and effort costs, future studies should utilize a broader range of effort costs including a no-effort condition.

The current work has significant implications for the study of personal control and effort. Across fields such as counseling, motivation science, and education, there is a common fundamental goal of enhancing intrinsic motivation to equip individuals to tackle effortful personal challenges. While it is often not possible or not desirable to alter the level of effort required to achieve a goal, it may be possible to enhance intrinsic motivation to meet effort requirements. Our results suggest that when effort requirements are high, bolstering the experience of personal control to boost intrinsic motivation may require the experience of real control in order to overcome effort costs.

Our study provided evidence that a context of physical effort costs may negate the benefits of making some choices. Future research should explore whether the cognitive effort demanded by more computationally difficult choices (e.g., Kool et al., 2010) undermines the positive benefits of choice. Additionally, given that developmental stage and psychopathology can impact willingness or ability to expend cognitive effort in decision making (Leykin et al., 2011; Luke et al., 2012; Westbrook et al., 2013), future research could examine how altered decision costs might influence the motivational outcomes of choice.

While the beneficial effects of personal control have been repeatedly demonstrated across domains from performance on simple tasks and educational activities to coping with stress (e.g., Patall, 2013; Murayama et al., 2015; Murty et al., 2015; Bhanji et al., 2016), many studies have operationalized personal control via low-cost, simple choices that may or may not offer actual control. Given that effort is a ubiquitous requirement for nearly all goal achievement, and given that differing levels of control may be required to enhance motivation under different effort costs, it is important to examine the conjoint effects of control and cost factors in decision making. Our results shed light on the subtleties of how these factors may interact, suggesting that when costs are high, mere choice and illusory control alone may not suffice to enhance motivation. Rather, under effort requirements, opportunities to exert real control may be necessary to boost motivation. At the same time, our results suggest that conditions of illusory control may be transformed by even intermittent occasions of actual control, suggesting that enriched perceptions of control rather than complete and total personal control may be sufficient to motivate.

## Author Contributions

HS-T and ET conceptualized and designed the study. Data collection and analysis was completed by HS-T under the supervision of ET. All authors contributed to the interpretation of experimental results. HS-T drafted the manuscript and ET and JR provided critical revisions. All authors have approved the final version of the manuscript.

## Conflict of Interest Statement

The authors declare that the research was conducted in the absence of any commercial or financial relationships that could be construed as a potential conflict of interest.

## References

[B1] Alloy L. B. Abramson L. Y. (1979). Judgment of contingency in depressed and nondepressed students: sadder but wiser? *J. Exp. Psychol. Gen.* 108 441–485. 10.1037/0096-3445.108.4.441 528910

[B2] Anderson C. J. (2003). The psychology of doing nothing: forms of decision avoidance result from reason and emotion. *Psychol. Bull.* 129 139–166. 10.1037/0033-2909.129.1.139 12555797

[B3] Beattie J. Baron J. Hershey J. C. Spranca M. D. (1994). Psychological determinants of decision attitude. *J. Behav. Decis. Mak.* 7 129–144. 10.1002/bdm.3960070206

[B4] Bhanji J. P. Delgado M. R. (2014). Perceived control influences neural responses to setbacks and promotes persistence. *Neuron* 83 1369–1375. 10.1111/j.1574-6976.2012.00333.x 25199702 PMC4169331

[B5] Bhanji J. P. Kim E. S. Delgado M. R. (2016). Perceived control alters the effect of acute stress on persistence. *J. Exp. Psychol.* 145 356–365. 10.1037/xge0000137 PMC4755928 26726915

[B6] Botti S. Iyengar S. S. (2004). The psychological pleasure and pain of choosing: when people prefer choosing at the cost of subsequent outcome satisfaction. *J. Person. Soc. Psychol.* 87 312–326. 10.1037/0022-3514.87.3.312 15382982

[B7] Botvinick M. M. Huffstetler S. McGuire J. T. (2009). Effort discounting in human nucleus accumbens. *Cogn. Affect. Behav. Neurosci.* 9 16–27. 10.3758/CABN.9.1.16 19246324 PMC2744387

[B8] Bown N. J. Read D. Summers B. (2003). The lure of choice. *J. Behav. Dec. Mak.* 16 297–308. 10.1002/bdm.447

[B9] Burger J. M. (1989). Negative reactions to increases in perceived personal control. *J. Pers. Soc. Psychol.* 56 246–256. 10.1037/0022-3514.56.2.246

[B10] Clark L. Lawrence A. J. Astley-Jones F. Gray N. (2009). Gambling near-misses enhance motivation to gamble and recruit win-related brain circuitry. *Neuron* 61 481–490. 10.1016/j.neuron.2008.12.031 19217383 PMC2658737

[B11] Cockburn J. Collins A. G. E. Frank M. J. (2014). A reinforcement learning mechanism responsible for the valuation of free choice. *Neuron* 83 551–557. 10.1016/j.neuron.2014.06.035 25066083 PMC4126879

[B12] Cordova D. I. Lepper M. R. (1996). Intrinsic motivation and the process of learning: beneficial effects of contextualization, personalization, and choice. *J. Educ. Psychol.* 88 715–730. 10.1037//0022-0663.88.4.715

[B13] Davis D. Sundahl I. Lesbo M. (2000). Illusory personal control as a determinant of bet size and type in casino craps games. *J. Appl. Soc. Psychol.* 30 1224–1242. 10.1111/j.1559-1816.2000.tb02518.x

[B14] Deci E. L. Ryan R. M. (1987). The support of autonomy and the control of behavior. *J. Pers. Soc. Psychol.* 53 1024–1037. 10.1037/0022-3514.53.6.1024 3320334

[B15] Fleming S. M. Thomas C. L. Dolan R. J. (2010). Overcoming status quo bias in the human brain. *Proc. Natl. Acad. Sci. U.S.A.* 107 6005–6009. 10.1073/pnas.0910380107 20231462 PMC2851882

[B16] Flowerday T. Schraw G. (2003). Effect of choice on cognitive and affective engagement. *J. Educ. Res.* 96 207–215. 10.1080/00220670309598810

[B17] Fujiwara J. Usui N. Park S. Q. Williams T. Iijima T. Taira M. (2013). Value of freedom to choose encoded by the human brain. *J. Neurophysiol.* 110 1915–1929. 10.1152/jn.01057.2012 23864380 PMC3798941

[B18] Garbarino E. C. Edell J. A. (1997). Cognitive effort, affect, and choice. *J. Consum. Res.* 24 147–158. 10.1086/209500

[B19] Gendolla G. H. E. Richter M. (2010). Effort mobilization when the self is involved: some lessons from the cardiovascular system. *Rev. Gen. Psychol.* 14 212–226. 10.1037/a0019742

[B20] Gourville J. T. Soman D. (2005). Overchoice and assortment type: when and why variety backfires. *Mark. Sci.* 24 382–395. 10.1287/mksc.1040.0109

[B21] Hafner R. J. White M. P. Handley S. J. (2016). The excess choice effect: the role of outcome valence and counterfactual thinking. *Br. J. Psychol.* 107 36–51. 10.1111/bjop.12120 25660197

[B22] Iyengar S. S. Lepper M. R. (2000). When choice is demotivating: can one desire too much of a good thing? *J. Pers. Soc. Psychol.* 79 995–1006. 10.1037/0022-3514.79.6.995 11138768

[B23] Katz I. Assor A. (2006). When choice motivates and when it does not. *Educ. Psychol. Rev.* 19 429–442. 10.1007/s10648-006-9027-y

[B24] Kool W. McGuire J. T. Rosen Z. B. Botvinick M. M. (2010). Decision making and the avoidance of cognitive demand. *J. Exp. Psychol. Gen.* 139 665–682. 10.1037/a0020198 20853993 PMC2970648

[B25] Kurniawan I. T. Guitart-Masip M. Dayan P. Dolan R. J. (2013). Effort and valuation in the brain: the effects of anticipation and execution. *J. Neurosci.* 33 6160–6169. 10.1523/JNEUROSCI.4777-12.2013 23554497 PMC3639311

[B26] Kurniawan I. T. Seymour B. Talmi D. Yoshida W. Chater N. Dolan R. J. (2010). Choosing to make an effort: the role of striatum in signaling physical effort of a chosen action. *J. Neurophysiol.* 104 313–321. 10.1152/jn.00027.2010 20463204 PMC2904211

[B27] Langer E. J. (1975). The illusion of control. *J. Pers. Soc. Psychol.* 32 311–328. 10.1037/0022-3514.32.2.311

[B28] Langer E. J. Rodin J. (1976). The effects of choice and enhanced personal responsibility for the aged: a field experiment in an institutional setting. *J. Pers. Soc. Psychol.* 34 191–198. 10.1037/0022-3514.34.2.191 1011073

[B29] Leotti L. Delgado M. R. (2014). The value of exercising control over monetary gains and losses. *Psychol. Sci.* 25 596–604. 10.1177/0956797613514589 24390827 PMC3970926

[B30] Leotti L. A. Cho C. Delgado M. R. (2015). “The neural basis underlying the experience of control in the human brain,” in *The Sense of Agency* eds Haggard P. Eitam B. (Oxford: Oxford University Press).

[B31] Leotti L. A. Delgado M. R. (2011). The inherent reward of choice. *Psychol. Sci.* 22 1310–1318. 10.1177/0956797611417005 21931157 PMC3391581

[B32] Leotti L. A. Iyengar S. S. Ochsner K. N. (2010). Born to choose: the origins and value of the need for control. *Trends Cogn. Sci.* 14 457–463. 10.1016/j.tics.2010.08.001 20817592 PMC2944661

[B33] Leykin Y. Roberts C. S. Derubeis R. J. (2011). Decision-making and depressive symptomatology. *Cogn. Ther. Res.* 35 333–341. 10.1007/s10608-010-9308-0 PMC3132433 21841849

[B34] Luke L. Clare I. C. H. Ring H. Redley M. Watson P. (2012). Decision-making difficulties experienced by adults with autism spectrum conditions. *Autism* 16 612–621. 10.1177/1362361311415876 21846664

[B35] Moller A. C. Deci E. L. Ryan R. M. (2006). Choice and ego-depletion: the moderating role of autonomy. *Pers. Soc. Psychol. Bull.* 32 1024–1036. 10.1177/0146167206288008 16861307

[B36] Murayama K. Matsumoto M. Izuma K. Sugiura A. Ryan R. M. Deci E. L. (2015). How self-determined choice facilitates performance: a key role of the ventromedial prefrontal cortex. *Cereb. Cortex* 25 1241–1251. 10.1093/cercor/bht317 24297329

[B37] Murty V. P. DuBrow S. Davachi L. (2015). The simple act of choosing influences declarative memory. *J. Neurosci.* 35 6255–6264. 10.1523/JNEUROSCI.4181-14.2015 25904779 PMC4405547

[B38] Patall E. A. (2012). “The motivational complexity of choosing: a review of theory and research,” in *Oxford Handbook of Motivation* ed. Ryan R. (New York, NY: Oxford University Press) 249–279.

[B39] Patall E. A. (2013). Constructing motivation through choice, interest, and interestingness. *J. Educ. Psychol.* 105 522–534. 10.1037/a0030307

[B40] Patall E. A. Cooper H. Robinson J. C. (2008). The effects of choice on intrinsic motivation and related outcomes: a meta-analysis of research findings. *Psychol. Bull.* 134 270–300. 10.1037/0033-2909.134.2.270 18298272

[B41] Pittman T. S. Davey M. E. Alafat K. A. Wetherill K. V. Kramer N. A. (1980). Informational versus controlling verbal rewards. *Pers. Soc. Psychol. Bull.* 6 228–233. 10.1177/07399863870092005

[B42] Reeve J. Nix G. Hamm D. (2003). Testing models of the experience of self-determination in intrinsic motivation and the conundrum of choice. *J. Educ. Psychol.* 95 375–392. 10.1037/0022-0663.95.2.375

[B43] Ritov I. Baron J. (1990). Status-quo and omission biases. *J. Risk Uncertain.* 5 49–61.

[B44] Rodin J. Langer E. J. (1977). Long-term effects of a control-relevant intervention with the institutionalized aged. *J. Pers. Soc. Psychol.* 35 897–902. 10.1037/0022-3514.35.12.897 592095

[B45] Rotter J. B. (1966). Generalized expectancies for internal versus external control of reinforcement. *Psychol. Monogr.* 80 1–28. 10.1037/h0092976 5340840

[B46] Ryan R. M. Deci E. L. (2000). Self-determination theory and the facilitation of intrinsic motivation, social development, and well-being. *Am. Psychol.* 55 68–78. 10.1037/0003-066X.55.1.68 11392867

[B47] Samuelson W. Zeckhauser R. (1988). Status quo bias in decision making. *J. Risk Uncertain.* 1 7–59. 10.1007/BF00055564

[B48] Suzuki S. (1997). Effects of number of alternatives on choice in humans. *Behav. Process.* 39 205–214. 10.1016/S0376-6357(96)00049-6 24896966

[B49] Suzuki S. (2000). Choice between single-response and multichoice tasks in humans. *Psychol. Rec.* 50 105–115.

[B50] Tricomi E. M. Delgado M. R. Fiez J. A. (2004). Modulation of caudate activity by action contingency. *Neuron* 41 281–292. 10.1016/S0896-6273(03)00848-1 14741108

[B51] Vohs K. D. Baumeister R. F. Schmeichel B. J. Twenge J. M. Nelson N. M. Tice D. M. (2008). Making choices impairs subsequent self-control: a limited-resource account of decision making, self-regulation, and active initiative. *J. Pers. Soc. Psychol.* 94 883–898. 10.1037/0022-3514.94.5.883 18444745

[B52] Wegener D. M. Wheatley T. (1999). Apparent mental causation. *Am. Psychol.* 54 480–492. 10.1037/0003-066X.54.7.480 10424155

[B53] Westbrook A. Kester D. Braver T. S. (2013). What is the subjective cost of cognitive effort? Load, trait, and aging effects revealed by economic preference. *PLoS ONE* 8:e68210 10.1371/journal.pone.0068210 PMC3718823 23894295

[B54] Yu R. Mobbs D. Seymour B. Calder A. J. (2010). Insula and striatum mediate the default bias. *J. Neurosci.* 30 14702–14707. 10.1523/JNEUROSCI.3772-10.2010 21048128 PMC6633627

