# Supplementary material for: Control and Effort Costs Influence the Motivational Consequences of Choice
Source: Front Psychol. 2017 May 3;8:675. doi: 10.3389/fpsyg.2017.00675 (PMC5413552; doi:10.3389/fpsyg.2017.00675)
Supplement: Supplementary file 1 [file Data_Sheet_1.docx]

**Supplementary Material**

**Supplementary Method**

Choice and Effort Conditions

The full set of conditions included two levels of effort costs (Low-Requirement and High-Requirement, as defined by the number of key presses required) and three different levels of control provided across choice conditions (Free-Choice, Restricted-Choice, and No-Choice, as defined by the level of control over effort costs conferred by the blaster options offered). In the No-Choice condition, a single blaster was offered individually without another option. In the Free- and Restricted-Choice conditions, two blasters were offered for participants to choose between. The key difference between these two choice conditions was that in the Free-Choice condition (Experiment 4 only), participants were offered a choice between one High- and one Low-Requirement blaster and were allowed to freely choose which they preferred to use, while in the Restricted-Choice condition, participants were offered a choice between two different colored blasters in the same effort category (e.g., a choice between two High-Requirement blasters), such that their choice actually had no effect on the amount of effort required to fill the charge bar. Thus, the Restricted-Choice condition offered only illusory control. Subtle blaster color categories represented the choice and effort conditions, with two similarly colored exemplar blasters in each category (See Figure 1). Having two exemplar blasters per condition allowed implementation of the Restricted-Choice condition with a choice between two blasters from the same effort category. Low-Requirement blasters required a random number of presses between 11 and 20 to fill the effort bar, while High-Requirement blasters required a random number of presses between 21 and 30.

All experimental choice and effort contingencies were imbued with some ambiguity in order to facilitate subjective inferences regarding perceptions of control. Specifically, three design features were implemented: (1) no explicit information was given to participants about the effort levels or the nature of the choice conditions; rather, participants’ only information about experimental conditions was acquired through playing the game; (2) blaster cues representing choice and effort conditions were organized into subtle color categories and the mapping of choice and effort contingencies onto the color scheme was also not explicitly stated; and (3) effort requirements (number of presses) were randomly drawn from ranges that were only subtly different between the Low- and High-Requirement conditions so that effort contingencies were somewhat uncertain. We expected that these ambiguities would facilitate subjective inferences regarding the level of control conferred by the choice conditions, particularly in the Restricted-Choice (illusory control) condition.

Task

See Figure 2 for a representative schematic of a single trial within the tasks. At the beginning of each trial, a blaster cue screen presented blaster options for the trial (two options for Choice conditions or a single blaster for the No-Choice condition) was displayed for an unlimited time until the participant responded to activate the indicated blaster. Next, a jittered fixation was displayed before the “charge bar” appeared so that participants could not exactly predict the charge bar onset, thus minimizing preemptive first presses. When the effort period began at the appearance of the charge bar, participants were to begin making fast repetitive key presses until the charge bar was filled. The charge bar was the same size for Low- and High-Requirement trials but filled at different proportions, depending on a pre-set number of presses required on a given trial. The number of required presses was not explicitly indicated to participants but could be implicitly estimated from the proportion of the bar filled with each press.

In order to standardize subjective difficulty, the task was individually calibrated to each participant’s performance on a pre-game training task (Knutson, *et al.,* 2000; Mangels, *et al.,* 2006), such that success rates were high across all conditions. The specific time allotted for the charge bar on a given trial was determined by the formula *c_t_* = *r*(*p_t_*)(1.1) + (*r_t_*)(1.1), where c_t_  is allotted charge time, *r* is the required number of presses for a given trial, and *r_t_* and *p_t_* are the participant’s average pre-game reaction time to make an initial key press, and the participant’s average pre-game time between successive presses, respectively. Average individual pre-game reaction time values (both *r_t_* and *p_t_*) were both multiplied by 110% so that the task would be challenging but not impossible for participants. If the participant successfully completed the required number of presses in the allotted time, then the blaster “fired” and a “pow” symbol was displayed for 500ms, followed by an alien with an “X” on its head, displayed for 1250ms, indicating the alien was successfully “blasted.” If the required presses were not completed in the allotted time, then a “No” symbol was displayed for 500ms, followed by an alien icon, displayed for 1250ms, indicating the alien escaped.

Stimuli Sets

All eight blasters were used in each experiment, either with all eight used within each participant (Experiments 3 and 4) or with a set of four stimuli used within each participant, counterbalanced such that all eight stimuli were used between participants (Experiments 1 and 2). See Figure 1 for an example of the blaster stimuli sets used in each experiment. Across all experimental stimuli sets, color-hue category (green vs. orange) represented choice conditions and color-value category (lightness vs. darkness) represented effort conditions; within these constraints, conditions were fully counterbalanced across color categories, such that for each experiment, conditions were mapped onto color categories to create four versions of blaster stimuli, used across participants. For example, in Experiment 3 light green blasters represented Choice, Low-Requirement for one participant’s game and No-Choice, High-Requirement for another participant’s game. Thus, we aimed to minimize the chances of differences (both within an experiment and between experiments) resulting from preference for one color blaster over another. In experiments in which a condition was not used (Choice in Experiment 1; High- Requirement in Experiment 2), one respective color category was excluded from each of the stimuli sets used in that particular experiment (e.g., in Experiment 1, only green *or* orange blasters were used for an individual participant’s set of stimuli) The specific mapping of condition to color category that was used in each experiment is detailed in the respective experiment.

**Supplementary Results**

Potential Effects of Learning

The task used in the present study was designed to minimize the role of learning such that learning the choice and effort cues would not be necessary for successful performance. Several aspects of the design minimized the role of learning in achieving successful performance: (1) participants were given substantial training on how to fill the “effort bar” before the task began (although they were not trained on the choice and effort cues), (2) the effort bar was displayed on screen during the trial showing participants in real-time how quickly they were filling the bar (3) the amount of time allotted for filling the bar across all conditions was determined by the same formula. Nevertheless, it is possible that participants learned the cues at different rates and that this affected success rates across the different conditions.

Our task was not designed to measure learning, and it is not possible to attribute unsuccessful trials to participants not having learned the effort contingencies because we cannot rule out the possibility that participants were simply not motivated to complete the trial. Thus, it might not be possible to assess learning on a trial-by-trial basis. However, we can address the possibility that learning, rather than motivation, may have influenced the differential performance rates across the conditions. To examine this possibility, we explored success rates across blocks (Fig S1) and within block 1, comparing the first 16 trials to the second 16 trials (Fig S2). Figure S1 shows that, for all effort conditions in all experiments, success rates were above 95% in the first block and either remained constant or diminished across successive blocks. Similarly, Figure S2 shows that, for all effort conditions in all experiments, success rates were well above 95% even in the first 16 trials of the games. For all effort conditions in all experiments, except for the High-Requirement condition in Experiment 1, success rates diminished from the first 16 trials of the game to the second 16 trials of the game. In the High- Requirement condition in Experiment 1, success rates increased slightly from 97.6% (*SD* = 7.2%) in the first 16 trials of the game to 98.3% (*SD* = 4.4%) in the second 16 trials of the game, however, this difference was not significant (*t*(36) = -.627, *p* = .535). From this pattern of results we can infer that participants learned how to successfully complete trials in all conditions very early in the games and that diminishing success rates across successive blocks were not due learning.


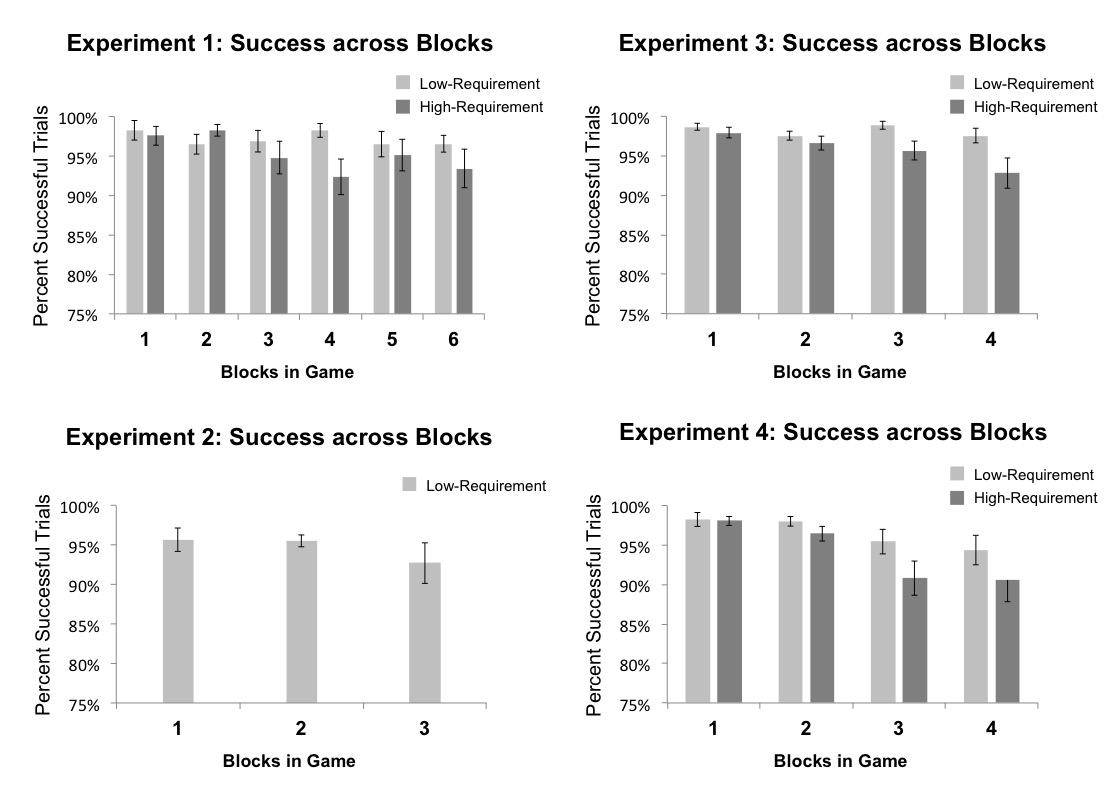


**Figure S1:**

Success rates across blocks of the game for all experiments.

**
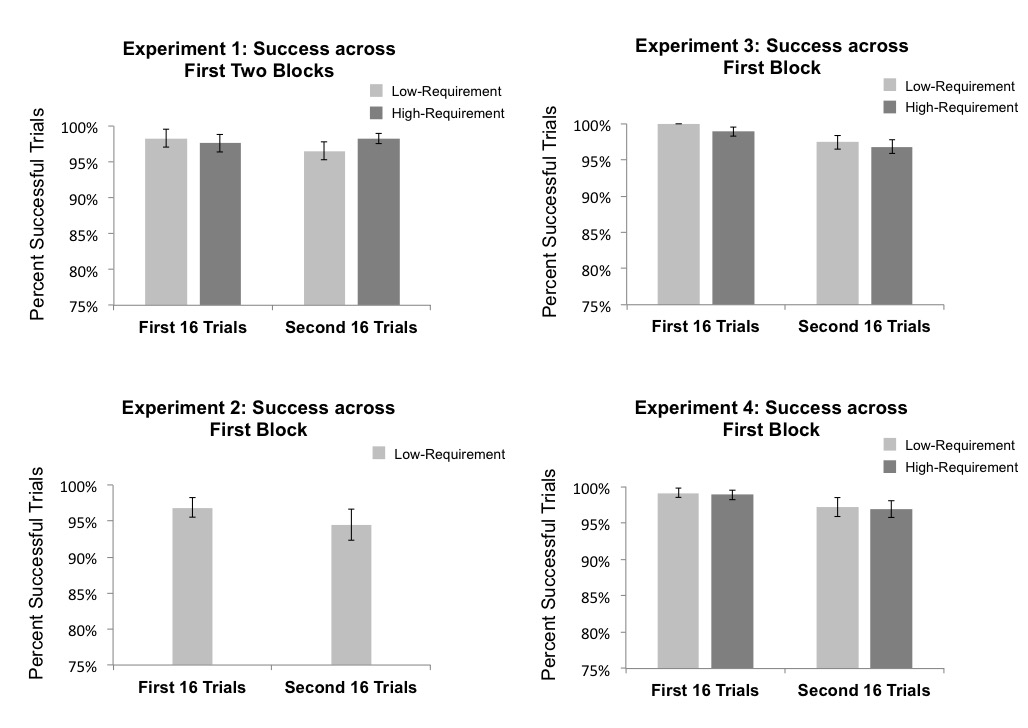
**

**Figure S2:**

Success rates across the first and second 16 trials of the game for all experiments.

**Supplementary References**

Knutson, B., Westdorp, A., Kaiser, E., & Hommer, D. (2000). FMRI visualization of brain activity during a monetary incentive delay task. *NeuroImage*, 12(1), 20–7. http://doi.org/10.1006/nimg.2000.0593

Mangels, J. A., Butterfield, B., Lamb, J., Good, C., & Dweck, C. S. (2006). Why do beliefs about intelligence influence learning success? A social cognitive neuroscience model. *Social Cognitive and Affective Neuroscience*, 1(2), 75–86. http://doi.org/10.1093/scan/nsl013
